# Editorial: Electromagnetic field theories of consciousness: opportunities and obstacles

**DOI:** 10.3389/fnhum.2023.1342634

**Published:** 2024-03-01

**Authors:** Tam Hunt, Mostyn Jones, Johnjoe McFadden, Arnaud Delorme, Colin G. Hales, Marissa Ericson, Jonathan Schooler

**Affiliations:** ^1^Department of Psychological and Brain Sciences, University of California, Santa Barbara, Santa Barbara, CA, United States; ^2^Independent Researcher, Philadelphia, PA, United States; ^3^Department of Chemistry, University of Surrey, Surrey, United Kingdom; ^4^University of California, San Diego, San Diego, CA, United States; ^5^Department of Physics, The University of Melbourne, Parkville, VIC, Australia; ^6^University of Southern California, Los Angeles, CA, United States; ^7^University of California, Santa Barbara, Santa Barbara, CA, United States

**Keywords:** electromagnetic fields, field theories of consciousness, philosophy of mind, psychophysical laws, EM field theories of consciousness

We are excited about the articles published on this Research Topic, “*Electromagnetic field theories of consciousness: opportunities and obstacles*,” appearing here for the first time as a Research Topic.

While the concept of an EM field theory of mind is not new – it was first proposed over 70 years ago – it is indeed a new development to see this level of interest in this type of solution for the infamous “hard problem” of consciousness, and of course “the easy problems” of consciousness too. In fact, that's one of the key features of EM field theories of consciousness: they can address both the broader philosophical and fundamental physics questions of consciousness, and also the nuts and bolts of how the brain works from moment to moment and day to day.

Our Research Topic was, in part, a celebration of the 30th anniversary of the game-changing “neural correlates of consciousness” concept, first proposed as part of Crick and Koch's 1990 “neurobiological theory of consciousness.” After now 33 years of research and theory-building, however, scholars in the science of consciousness are perhaps not much closer to a widely accepted theory of consciousness.

An electromagnetic (EM) field theory of consciousness attempts to explain the nature of consciousness and its relationship to matter in terms of fundamental EM fields and their dynamics. EM field theories view brain waves (delta, theta, etc.) and related EM fields as causally potent and functionally relevant to consciousness and the workings of the brain. EM field theories are a promising and growing subset of consciousness theories.

These theories originally emerged because they drew on considerable experimental evidence and provided potential solutions to traditional neuroscience's well-known problems. For example, how does the unity of consciousness arise from the functioning of billions of neurons and glia?

It is worth noting that most physicalist theories of consciousness boil down to a type of EM field theory of consciousness, whether or not this is acknowledged. This is the case because the atomic basis of the material comprising our brains, our bodies, and our biosphere is intrinsically electromagnetic. Other fundamental forces – gravity and the strong and weak nuclear forces – are likely not relevant to the dynamics of consciousness. In this manner, all of the physical dynamics that affect consciousness are ultimately various kinds of EM field dynamics, so even when a theory doesn't mention EM fields specifically, and if it is a physical theory of consciousness, then it will be based in some manner on EM fields.

The specific role of EM fields in the brain has been debated for many years, with some scholars maintaining the view that they are largely or entirely epiphenomenal – like the proverbial train whistle on a steam-powered locomotive – and other scholars viewing them as integral to the workings of consciousness. We are now at a point where experiments and data are being brought to bear to resolve this debate.

Our anchor article for this Research Topic was Hunt and Schooler's 2019 paper, “The easy part of the Hard Problem: A resonance theory of consciousness.” The General Resonance Theory (GRT) of consciousness, described in that paper, may be viewed as a type of electromagnetic theory of consciousness and posits that electromagnetic (EM) fields may be the primary seat of consciousness. As such, the dynamics of these fields become the measurable dynamics of consciousness. This remains a hypothesis but experiments are being conducted in various labs around the world to test this exciting hypothesis. The various papers in this issue shed light on this hypothesis and related ideas surrounding EM field theories.

MacIver's paper, “*Consciousness and Inward Electromagnetic Field Interactions*,” provides insights into how electromagnetic fields generated by neuronal membranes might be crucial for consciousness. The paper addresses early criticisms of EM field theories and explores the use of non-linear dynamic analyses of EEG recordings to track consciousness levels. MacIver proposes an inward view of EMF energy “clouds,” suggesting that EM fields focused inward to the brain could provide stronger ephaptic connections to neural circuits and thus be causal, contrary to early critiques of EM field theories. This paper is significant for the Research Topic as it supports the idea that EM fields likely play a key role in mind-brain integration, and offers a new perspective on interpreting EEG data in the context of consciousness.

Keppler's paper, “*Building blocks for the development of a self-consistent electromagnetic field theory of consciousness*,” aims to assemble the foundational elements for creating a fundamental electromagnetic field theory of consciousness. It emphasizes the quantum electrodynamics vacuum state as a vibrant energy source, termed the zero-point field (ZPF), which is central to all electromagnetic phenomena. The paper theorizes that the brain functions as a resonant oscillator, selectively coupling to specific ZPF modes to compose specific phenomenal states. This theory posits consciousness as a result of the brain's interaction with ZPF modes, highlighting the significance of neurotransmitter-ZPF interactions for future research.

Young, Robbins et al.'s paper, “*From micro to macro: the combination of consciousness*,” explores the concept of consciousness extending beyond the individual to a collective level. It examines the synchronization of neuronally generated EM fields between individuals, proposing a model where individual agents may merge into a hierarchical cognitive system. The paper utilizes the axioms and conjectures of General Resonance Theory to describe this phenomenon of interpersonal resonant combination, suggesting that synchronized EM fields through behavioral interactions can optimize information flow and alter the conscious states of the agents involved. This research extends EM field approaches by proposing a physical basis for “group consciousness” and its empirical investigation.

Kitchener and Hales' paper, “*What neuroscientists think, and don't think, about consciousness*,” discusses the prevailing approach of neuroscientists toward consciousness, primarily focusing on its generation and characteristics, without a consensus on its underlying mechanism. It emphasizes the integral role of neurons and electromagnetic fields in brain functioning, underscoring the complexity of electromagnetic phenomena from the atomic level upwards in the brain. This research adds to the EM field theories of consciousness by highlighting the fundamental physics of neurons and glial cells in the brain, suggesting that a deeper investigation into the electromagnetic fields at the cellular scale could offer insights into the mechanisms of consciousness.

Winters' paper, “*The temporally-integrated causality landscape: reconciling neuroscientific theories with the phenomenology of consciousness*,” presents the Temporally-Integrated Causality Landscape (TICL) as a framework to understand consciousness. It compares and contrasts TICL with other neuroscientific theories like Integrated Information Theory, GRT, and Global Neuronal Workspace Theory, emphasizing the importance of electromagnetic forces in neural causality. The paper contributes to the electromagnetic field theories of consciousness by exploring the spatial-temporal dynamics of brain activity and their relation to conscious experiences, proposing a more comprehensive approach to understanding consciousness in neurological terms.

The Young, Hunt et al. paper, “*The slowest shared resonance: a review of electromagnetic field oscillations between central and peripheral nervous systems*,” examines the role of EM field oscillations in both central and peripheral nervous systems. It explores the principle of the Slowest Shared Resonance (SSR) within GRT, positing that consciousness arises from the combination of micro- to macro-consciousness in coupled field systems, determined by the slowest common denominator frequency. This paper contributes to the Research Topic by suggesting a spatiotemporal hierarchy of brain-body shared resonance systems and supports the principle of SSR within EM field theories of consciousness.

Hales and Ericson's paper, “*Electromagnetism's bridge across the explanatory gap: how a neuroscience/physics collaboration delivers explanation into all theories of consciousness*,” focuses on integrating neuroscience and fundamental physics to address the “explanatory gap” in consciousness research. It argues that the brain, as an electromagnetic field object, can be understood through the standard model of particle physics, suggesting that all theories of consciousness are essentially interpretations of specific EM field behaviors in brain tissue. This interdisciplinary approach aims to provide a unified explanation applicable to all theories of consciousness, exploring how subjectivity might emerge from electromagnetic fields.

Ward and Guevara's paper, “*Qualia and phenomenal consciousness arise from the information structure of an electromagnetic field in the brain*,” explores the physical substrate for subjective, phenomenal consciousness (P-consciousness). It proposes that the electromagnetic (EM) field generated by the brain's electrical charges serves as this substrate. The paper posits that a part of the thalamus in mammals generates this critical EM field, which is structured by emulating information from external and internal sources, forming the basis of qualia experienced in P-consciousness. This research contributes to EM field theories by suggesting how the brain's EM fields may structure the experience of consciousness.

Bond's paper, “*The contribution of coherence field theory to a model of consciousness*,” delves into the emerging paradigm in neuroscience that views resonance as central to consciousness. It discusses the role of oscillating flows within the brain's electric field in producing mind from matter and explores how vibrations in nanoscale atomic structures and photonic waves may contribute to consciousness. The paper touches on the “binding problem” in consciousness theory, questioning how trillions of atoms and billions of cells integrate to produce a unified medium of awareness. Bond also investigates how EM fields within neurons influence signal transmission, surpassing explanations based solely on ion diffusion. The paper's relevance lies in its exploration of how light interactions with biological systems and internal EM fields in the brain could contribute to consciousness, aligning with the Research Topic's focus on EM fields.

Hunt and Jones “*Fields or firings? Comparing the spike code and the electromagnetic field hypothesis*,” proposes that EM fields, from the local to the global, may be the primary seat of consciousness in the brain. It contrasts this hypothesis with the conventional spike code approach that focuses on synaptic firing as the basis for consciousness. The paper posits that while neurons and synaptic transmissions are necessary for consciousness, they are not sufficient to explain its complexity. It argues that consciousness arises from the intricate interplay between neuronal activities and EM fields, suggesting that these fields, rather than being epiphenomenal, play a central role in the emergence and unification of conscious cognition. The authors highlight the importance of EM fields in various cognitive processes, including memory and perception, and call for further research in this area. They present various sources of evidence that oscillating neural EM fields may make firing in neural circuits oscillate, and these oscillating circuits may help unify and guide conscious cognition.

“*Consciousness: Meat or EMF?*” by McFadden challenges conventional theories of consciousness that rely on the brain's neuronal matter, proposing instead that the substrate of consciousness is the brain's electromagnetic field. The paper critiques existing theories, showing how EM field theories provide novel insights into consciousness and potentially offer a route toward building artificial consciousness. It distinguishes between intelligence and consciousness, arguing that EM theories account for the emergence of consciousness through natural selection and the brain's neural activity. This paper contributes significantly to the Research Topic by offering a comprehensive examination of EM theories against established criteria and by discussing the evolutionary aspects of consciousness in relation to electromagnetic fields.

“*Electromagnetic-field theories of qualia: can they improve upon standard neuroscience?*” by Jones and Hunt, explores the potential of EM field theories in explaining qualia, the subjective aspects of consciousness like colors, pains, and emotions, which have been challenging for standard neuroscience to fully account for. The authors review various EM field theories of qualia of how our various qualia arise, assessing their strengths and weaknesses, and contrasting them with traditional synaptic neuroscience approaches. They focus on three key problems: identifying neural correlates of the various qualia, integrating qualia into a unified perceptual experience, and addressing the “hard problem” of consciousness, namely the metaphysical relationship between neural events and qualia. The paper suggests that EM field theories, while still in development, could offer promising avenues for better understanding consciousness and qualia, potentially improving upon the explanations provided by standard neuroscience.

Lacalli's paper, “*Consciousness and its hard problems: separating the ontological from the evolutionary*,” focuses on the role of evolution in theories of consciousness. It introduces the concept of a “consciousness machine” to explore how ontology and evolution contribute to consciousness. The paper examines whether consciousness originates from electromagnetic field effects or neural connectivity and information flow. It also discusses the evolution of consciousness and agency, suggesting that agency might be more a developmental than evolutionary process. The paper explores the emergence of consciousness and behavior links, suggesting a divide between phenomenal experience and agency in developmental and evolutionary timescales. The author concludes that understanding consciousness involves both easy problems, like the neurocircuitry innovations for consciousness, and hard problems, like the ontological basis of subjective experience.

The final paper, Gómez-Emilsson and Percy “*Don't forget the boundary problem! How EM field topology can address the overlooked cousin to the binding problem for consciousness*,” explores the “boundary problem” in theories of consciousness, an issue often overshadowed by the more widely discussed binding problem. The authors propose that EM field topology could be a key to understanding how distinct boundaries of consciousness are formed. They argue that while existing theories focus on how various experiences are unified into a single first-person perspective (the binding problem), they often neglect the question of why these unified experiences have specific spatial and temporal boundaries (the boundary problem). By examining EM field theories, the paper suggests that topological segmentation within EM fields could conceptually and empirically address this boundary problem, offering a novel perspective in consciousness studies.

In closing, it is our strong hope that these papers extend discussion and research into EM field theories for many years to come — and may even lead to a more widely accepted set of solutions to the Hard Problem as well as the easy problems of consciousness.

## Author contributions

TH: Writing—original draft. MJ: Writing—review & editing. JM: Writing—review & editing. AD: Writing—review & editing. CH: Writing—review & editing. ME: Writing—review & editing, Supervision, Conceptualization, Project administration. JS: Writing—review & editing, Supervision, Conceptualization, Project administration.

